# Structural Insight into the Mechanism of PALB2 Interaction with MRG15

**DOI:** 10.3390/genes12122002

**Published:** 2021-12-17

**Authors:** Jennifer Redington, Jaigeeth Deveryshetty, Lakshmi Kanikkannan, Ian Miller, Sergey Korolev

**Affiliations:** Edward A. Doisy Department of Biochemistry and Molecular Biology, Saint Louis University School of Medicine, St. Louis, MO 63104, USA; jenny.redington@slu.edu (J.R.); jaigeeth.deveryshetty.1@health.slu.edu (J.D.); lakshmi.kanikkannan@slu.edu (L.K.); ian.miller@health.slu.edu (I.M.)

**Keywords:** genome maintenance, recombination mediator, crystal structure, DNA repair, transcription complex, homologous recombination, protein-protein interaction, cancer mutations

## Abstract

The tumor suppressor protein partner and localizer of BRCA2 (PALB2) orchestrates the interactions between breast cancer susceptibility proteins 1 and 2 (BRCA1, -2) that are critical for genome stability, homologous recombination (HR) and DNA repair. PALB2 mutations predispose patients to a spectrum of cancers, including breast and ovarian cancers. PALB2 localizes HR machinery to chromatin and links it with transcription through multiple DNA and protein interactions. This includes its interaction with MRG15 (Morf-related gene on chromosome 15), which is part of many transcription complexes, including the HAT-associated and the HDAC-associated complexes. This interaction is critical for PALB2 localization in actively transcribed genes, where transcription/replication conflicts lead to frequent replication stress and DNA breaks. We solved the crystal structure of the MRG15 MRG domain bound to the PALB2 peptide and investigated the effect of several PALB2 mutations, including patient-derived variants. PALB2 interacts with an extended surface of the MRG that is known to interact with other proteins. This, together with a nanomolar affinity, suggests that the binding of MRG15 partners, including PALB2, to this region is mutually exclusive. Breast cancer-related mutations of PALB2 cause only minor attenuation of the binding affinity. New data reveal the mechanism of PALB2-MRG15 binding, advancing our understanding of PALB2 function in chromosome maintenance and tumorigenesis.

## 1. Introduction

DNA is constantly damaged by genotoxic factors and intracellular metabolic processes. One of the intrinsic sources of chromosome breaks and replication stress in dividing cells is collisions between transcription and replication complexes [1,2]. Homologous recombination (HR) is the major non-mutagenic mechanism of chromosome break repair in dividing cells [3,4]. Breast cancer susceptibility proteins 1 and 2 (BRCA1 and -2) are positive regulators of HR [5,6,7]. Partner and localizer of BRCA2 (PALB2) was discovered as a protein forming stable complexes with BRCA2 and found to be responsible for the localization and stability of BRCA2 [8]. BRCA1 acts at the initial stage of damage recognition [9]. It interacts with PALB2, bringing BRCA2 to the break site together with RAD51 recombinase. In addition to break repair, these proteins are important for the repair of stalled or stressed replication forks [10,11,12]. PALB2 binds numerous cellular factors important for the regulation of DNA repair under various damage conditions [13,14]. Among other factors, PALB2 interacts with nucleosomes and with transcription factors tethering DNA repair machinery to chromatin and connecting it with transcription. The latter is achieved through interaction with the transcription factor MRG15 (Morf-related gene on chromosome 15), which is a part of many transcriptional regulator complexes such as the HAT-associated Tip60/NuA4 complex and the HDAC-associated Sin3S/Rpd3S complex [15,16,17,18]. These and other MRG15 complexes are critical for cell proliferation, embryonic development, DNA damage repair, and alternative splicing [15,17,18,19,20,21,22,23,24,25,26,27,28,29]. The association of PALB2 with MRG15 leads to the recruitment of HR machinery to actively transcribed genes, where conflicts between transcription and replication lead to frequent DNA damage even in the absence of genotoxic factors [18]. In order to define the mechanism of PALB2 interaction with transcription complexes, we solved the crystal structure of the MRG15 MRG domain bound to a PALB2 peptide. The structure revealed an extended interaction interface around the conserved MRG-binding FxLP motif corresponding to a nanomolar affinity. The interaction resembles complexes with MRG15 and several other transcription factors. The role of key interaction amino acids and known cancer-associated PALB2 mutations was assayed through solution binding studies.

## 2. Materials and Methods

### 2.1. Protein Expression and Purification 

The MRG15 MRG domain, encompassing amino acids 155–323, was cloned into the pET28b^+^-based pSMT3 vector (provided by Dr. R. A. Kovall, University of Cincinnati, Cincinnati, OH, USA) containing a N-terminal 6xHis-SUMO tag via the Gibson assembly protocol. pSMT3-MRG15^155−323^ was transformed into BL21* *Escherichia coli* cells (Thermo Fisher Scientific, Waltham, MA, USA). Cultures were grown in Terrific broth media to an OD_600_ of 1.6, and protein expression was induced with 0.2 mM IPTG at 16 °C overnight. Cells were collected by centrifugation at 4000 rpm for 30 min at 4 °C and resuspended in lysis buffer composed of Buffer A (25 mM HEPES pH7.5, 1 M NaCl, 10% glycerol, 1 mM TCEP, 2 mM CHAPS [Thermo Fisher Scientific; VWR International, Radnor, USA; Gold Biotechnology, Inc., Olivette, MO, USA; MilliporeSigma, St. Louis, MO, USA]) with the addition of 0.1% Brij-35, 1 mM PMSF and 15 mM imidazole. Cells were lysed with 0.25 mg/mL lysozyme for 20 min at 4 °C, then sonicated and centrifuged at 16,000 rpm for 30 min at 4 °C. Supernatant was bound to 8 mL Ni-NTA resin (Takara Bio Inc., Kusatsu, Japan). Beads were washed with 200 mL of lysis buffer, followed by a wash with 50 mL buffer A with decreased NaCl concentration (0.1 M NaCl). Subsequently, beads were washed by 50 mL of buffer A adjusted to 40 mM imidazole and finally by 20 mL of Buffer A with 100 mM imidazole. The protein was eluted by Buffer A with 200 mM imidazole. The SUMO tag was cleaved with Ulp1 protease digestion while dialyzing against Buffer A without imidazole overnight at 4 °C. The protein solution was applied to a Ni NTA column and protein was collected in a flowthrough fraction. Protein was then stored at −20 °C.

Four PALB2 fragments encompassing amino acids 564–620, 579–620, 597–630, and 597–674 were cloned into pMCSG7 plasmids [30] with the N-terminal 6xHis tag and expressed as above. Cells were lysed as described above, suspended in 8 M urea buffer and centrifuged at 16,000 rpm for 40 min. Supernatant was passed through a Superflow Ni-NTA resin equilibrated with 8 M urea. Protein bound Ni-NTA beads were washed sequentially with buffers containing 8 M, 4 M, 2 M, 1 M urea in Buffer B and eluted with 40 mL of Buffer C (25 mM HEPES pH 7.5, 10% glycerol, 2 mM CHAPS, 300 mM NaCl).

### 2.2. Pulldown Assay

Ni-NTA beads (200 µL of 50% slurry) were equilibrated with Buffer B (25 mM HEPES pH 7.5, 10% glycerol, 2 mM CHAPS and 300 mM NaCl). 1 mL of PALB2 peptide at concentration 0.1 mg·mL^−1^ was bound to Ni-NTA beads, and excess peptides were washed off with Buffer B (1 mL). A total of 1 mL of 0.1 mg·mL^−1^ MRG15^155−323^ was added to the beads and incubated for 30 min. Beads were washed 3 times with Buffer B with 20 mM imidazole. Protein complexes were eluted with Buffer B with 250 mM imidazole. MRG15^155−323^ alone, mixed with Ni-NTA beads without any peptide bound, was used as control to eliminate non-specific binding. Samples were analyzed by SDS-PAGE.

### 2.3. Crystallization and Structure Determination

Equal volumes of MRG15^155−323^ and 6xHis-PALB2^597−630^ at a concentration of 5 mg·mL^−1^ each were mixed. The buffer was then exchanged to Buffer B and the complex was concentrated to 10 mg·mL^−1^ using 15 mL Centricon filters with a 3 kDa MWCO. Crystallization conditions were screened using a Phoenix crystallization robot (Art Robbins Instruments, Sunnyvale, CA, USA) using commercial screens (Hampton Research, Aliso Viejo, USA; Molecular Dimensions, Maumee, OH, USA) in 96-sitting drop Intelli-Plates (Art Robbins Instruments) with 3 different protein-to-buffer volume ratios for each condition. A buffer with 2.5 M ammonium sulfate and 0.1 M Tris, pH 8.5, yielded diffraction-quality crystals. Diffraction was collected using the departmental X-ray facility with a Rigaku MMX-007 X-ray generator (1.2 kW), VariMax-HF optics, Raxis-IV++ detector and X-stream cryocooling system, and data were processed and scaled using the HKL2000 program [31] to 2.7 Å resolution. The structure was solved and refined using the Phenix program suite [32,33]. A molecular replacement solution was obtained using 2AQL coordinates [34]. PALB2 peptides were manually modeled into the electron densities. The structure was refined using torsion NCS restraints applied to MRG domains only. Data collection and refinement statistics are shown in Table 1. Coordinates were deposited into the Protein Data Bank [35] with PDB ID 7S4A. Protein interaction interfaces were analyzed with the PISA program [36].

### 2.4. Binding and Competition Assays

Fluorescence anisotropy binding and competition assays were performed using black 384-well plates (Corning Inc., Corning, NY, USA) and a Synergy Neo2 plate reader (BioTek, Winooski, VT, USA). Binding assays were conducted by serially diluting the MRG15 fragment from 10 µM to 0.6 nM. A 40 µL solution of 20 µM MRG was serially diluted in 20 µL of 25 mM HEPES pH8.0, 1 mM TCEP, 10% DMSO, and 200 mM NaCl. A total of 20 µL of a 20 nM FAM-PALB2^597−630^ solution was then added to each well for a final reaction volume of 40 µL and a final PALB2 concentration of 10 nM. The plate was shaken for 2 min and incubated for 15 min. Fluorescence anisotropy was measured with 485/20 nm excitation and 528/20 nm emission using Gen5.0 (BioTek) software. K_d_ was calculated with GraphPad Prism software by fitting the data with a non-linear regression analysis using a standard four-parameter logistic equation to identify K_d_.

(1) $$y=y_{min}+\frac{y_{max}\;–\;y_{min}}{1+10^{\left(logEC50-X\right)\;x\;n\;}}$$

where *y_min_* and *y_max_* are the minimum and maximum anisotropy values, *X* represents the log concentration of protein, n represents the Hill slope, and EC50 is equal to K_d_. R^2^ is determined by the Prism software by computing the sum of the squares of the distances of the points from the best-fit curve determined by a nonlinear regression model.

For competition assays, mutant PALB2^597−630^ peptides (Genscript Biotech, Piscataway, NJ, USA) were dissolved in DMSO and serially diluted from 10 µM to 0.6 nM in 20 µL of 25 mM HEPES pH 8.0, 1 mM TCEP, 10% DMSO, and 200 mM NaCl. A total of 40 nM MRG15 was mixed with 10 nM PALB2-FAM and incubated for 15 min at room temperature (RT). 20 µL of the MRG15 with FAM-PALB2 was then added to each well, resulting in a final concentration of 5 nM MRG15 and 20 nM FAM-PALB2. The total reaction volume was 40 µL. The plate was then shaken for 2 min and incubated for 15 min before the fluorescence anisotropy was measured as above. The inhibition constant K_i_ was calculated similarly to K_d_ (above).

## 3. Results

### 3.1. Identification of the MRG15-Binding Region of PALB2

**Figure 1:**
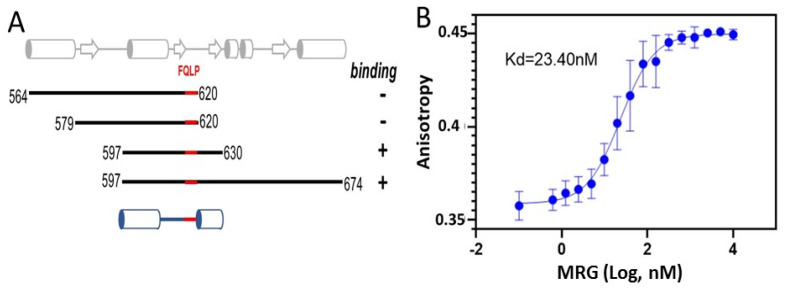
**MRG interaction with PALB2.** (**A**) Preliminary analysis of MRG binding by purified His-tagged PALB2 peptides represented schematically as black lines with numbers for N- and C-terminal amino acids shown at the ends and with the FQLP motif highlighted in red. Panel on top shows predicted secondary structure elements (α-helixes as cylinders and β-strands as arrows). Panel on the bottom shows secondary structure elements observed in a crystal structure. (**B**) Anisotropy isotherm of FAM-PALB2^597−630^ peptide titration of MRG.

Proteins interact with the MRG domain through a conserved FxLP motif [37,38,39]. All known structures of MRG complexes reveal significant contributions to the binding of extended regions up- and downstream of the FxLP motif. Flanking regions have low sequence and structural conservation, making it challenging to predict which regions of PALB2 around the FQLP sequence motif (amino acids 612–615) are involved in the interaction. To identify flanking regions important for binding, we isolated several PALB2 peptides of sequence regions 564–620, 579–620, 597–630, and 597–674 with 6xHis tags at N-termini and tested them for interactions with MRG15 in pull-down assays on Ni-NTA agarose. Results of this preliminary qualitative experiment suggested that both N- and C-terminal regions strongly contribute to MRG binding and that the peptide 597–630 is sufficient for the formation of a stable complex (Figure 1A). The binding affinity of this peptide to MRG15 was measured using the polarization anisotropy method with fluorescein-labeled PALB2^597−630^ peptide (FAM-PALB2, Genscript Biotech, Piscataway, NJ, USA). An estimated K_d_ = 23.4 ± 5 nM (Figure 1B) corresponds to strong binding and is similar to the value obtained using isothermal calorimetry analysis [38]. It is also characteristic for other MRG binders, e.g., the MRG-Pf1 MBD complex [37,38,39,40].

### 3.2. Crystal Structure of an MRG15 Complex with PALB2

**Figure 2:**
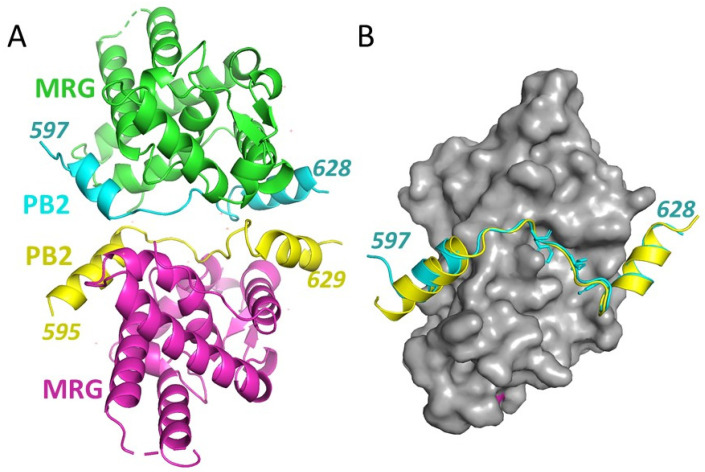
**Crystal structure of MRG PALB2 complex.** (**A**) Cartoon representation of two complexes in an asymmetric unit, with MRG chains shown in green and magenta and PALB2 peptides in cyan and yellow. N- and C-terminal amino acids modeled for each PALB2 peptide are shown. (**B**) Two complexes were superimposed by the MRG subunits (shown in the grey surface representation), resulting in the overlap of two PALB2^597−630^ peptides.

Crystals of the MRG15 MRG domain bound to the PALB2^597−630^ peptide were obtained, and a structure of the MRG15-PALB2 complex was solved by the molecular replacement method using the known MRG domain structure. An additional unoccupied 2Fo-Fc electron density was present next to the modelled MRG domains and was used to model PALB2 peptides. There are two complexes per asymmetric unit (Figure 2A).

Refinement was performed using torsion NCS restraints applied to MRG domains only. In both complexes, almost the entire peptide encompassing amino acids 597–626 was readily modeled. Only the last four amino acids of the PALB2 peptide are disordered. Peptides form extensive contacts with MRG similarly in both complexes (Figure 2B), with total contact areas of 1153 A^2^ and 1114 A^2^ (Figure 1) corresponding to a theoretical ΔG of −15 and −14 kcal/mol, correspondingly, for each complex, reflecting the strong interaction observed in solution studies. The conformations of the PALB2^597−630^ peptides in both complexes are almost identical, with a small difference at the N-terminal ends that can be attributed to different crystal packing environments.

**Figure 3:**
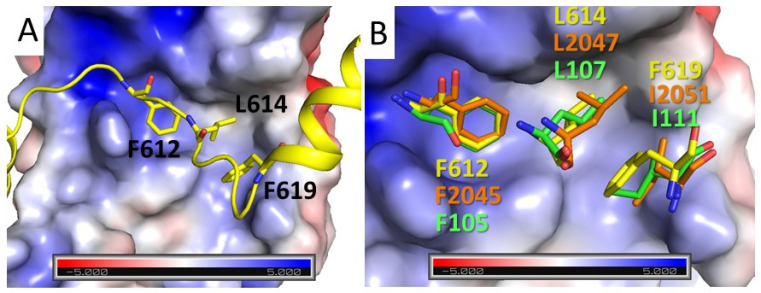
**Hydrophobic interactions of the core signature motif.** (**A**) Cartoon representation of PALB2 peptide in yellow with the conserved hydrophobic amino acids of the FxLP motif interaction with MRG shown in the surface representation and color-coded according to surface electrostatic potential. (**B**) Conformation comparisons of three hydrophobic amino acids of the FxLPxxФ motif from three different MRG-interacting peptides with carbon atoms of PALB2 shown in yellow, of 2N1D shown in green, and of 2INE in orange. All complexes were superimposed by MRG subunits shown as in panel A.

Within the conserved MRG-binding motif, amino acids Phe612, Leu614 and Pro615 form hydrophobic interactions with the MRG15 pocket that are identical to those described for other MRG-binding peptides (Figure 3A). Notably, the third hydrophobic chain of Phe619 is inserted into the extension of this pocket next to the side chain of Leu614 (Figure 3) and strongly contributes to binding, comparable to that of Phe612 and Leu614 (as shown below in Section 3.3). Examination of other known complexes of MRG revealed similar hydrophobic interactions in all known MRG-binding peptides with known structures (Figure 3B). Despite the spatially closed positioning of the third hydrophobic side chain (Phe619) to that of the conserved leucine in the FxLP motif, its position in the sequence varies, making it difficult to identify as a part of the conserved motif based on sequence alignment. Phe619 in PALB2 and Ile2051 in ASH1L (PDB: 6INE) [41] are separated from the conserved proline by 3 amino acids, while the corresponding Ile111 in MRGBP (PDB:2N1D) [38] is separated by 2 amino acids. Therefore, an MRG-binding conserved motif signature can be further refined as FxLP(x)_2–3_Φ, where Φ is a hydrophobic amino acid.

**Figure 4:**
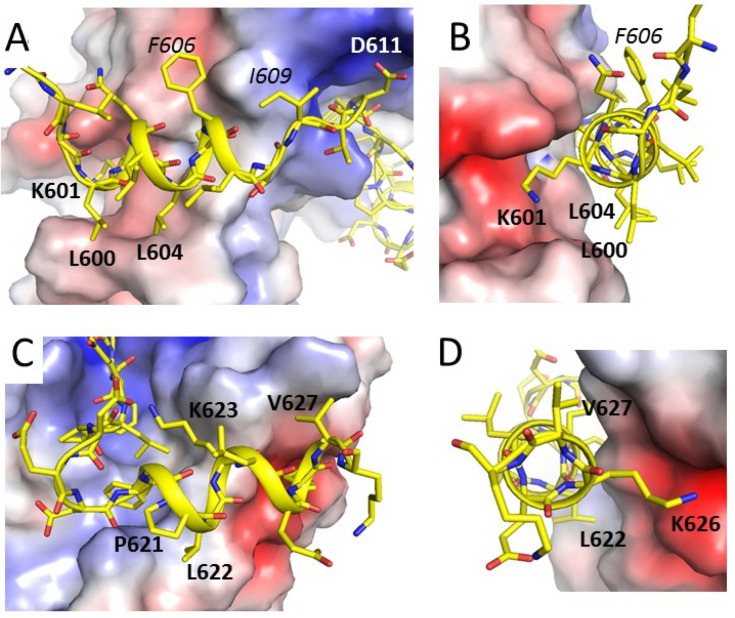
**Interactions of flanking helixes with MRG.** (**A**,**B**) Orthogonal views of αH1, shown in cartoon and stick representation, and interactions with the MRG domain shown in surface representation and color-coded accordingly to electrostatic potential; (**C**,**D**) two orthogonal views of αH2.

The PALB2 peptide is extended across the MRG surface, with 2 helices formed at amino acids 600–607 (αH1) and 619–628 (αH2) (Figure 2B and Figure 4) which bind at the opposite sides of MRG. Both helices form strong hydrophobic interactions with MRG hydrophobic cavities, while polar interactions by Lys601 and Lys626 anchor the helices at the N- and C-terminal ends (Figure 4). Asp611 between the αH1 and the FQLP motif interacts with a negatively charged area of MRG at the point where the peptide is bent around the MRG surface. αH1 is positioned in the shallow cavity of MRG and forms several hydrophobic contacts on each side of the helix by Leu600 and Leu 604 on one side and by Phe606 and Ile609 on the opposite side. Lys601 forms both hydrophobic and polar interactions with MRG. Similarly, αH2 binds a hydrophobic interface of MRG through Leu622 and Val627 and the aliphatic parts of Lys623 and Lys626 (Figure 4C,D). The side chain amino group of Lys626 is positioned at the negatively charged area of MRG. Lys623 forms a polar interaction with the carboxy group of Leu614 and stabilizes a bent conformation between the FQLP peptide and αH2.

### 3.3. Contribution to MRG Binding of PALB2 Conserved Amino Acids and Amino Acids Mutated in Cancer Patients

**Figure 5:**
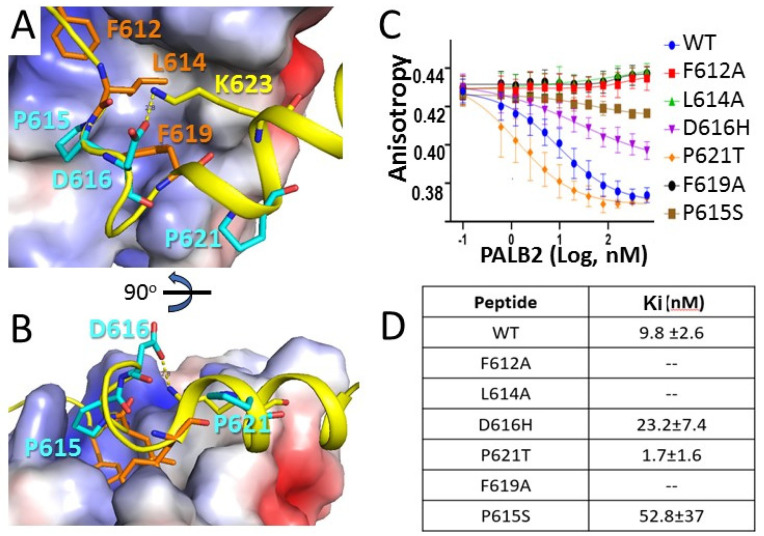
**Contribution of conserved amino acids and cancer-associated inherited mutations to the PALB2 interaction with MRG15.** (**A**,**B**) Two orthogonal views of the PALB2 interaction with MRG15, highlighting the residues identified in cancer patients shown in cyan and the binding hydrophobic core shown in orange. (**C**) Displacement of WT FAM-PALB2 peptide at 10 nM concentration from MRG with titration by mutant PALB2 peptides. (**D**) Dissociation constants Ki for each mutant calculated from titration isotherms shown in (**C**).

Mutations of several residues in the MRG-binding region of PALB2 were found in breast cancer patients [13,42,43,44], including Asp616His, Pro615Ser and Pro621Thr mutations (Figure 5A,B). We analyzed the effect of these mutations on PALB2 interaction with MRG by a competition assay (Figure 5C,D). An MRG complex with 10 nM WT PALB2 peptide was formed, and the comparative affinity of mutants (K_i_) was assayed by the dissociation rate of FAM-PALB2 from MRG15 upon titration by a mutant peptide. In addition, the contribution of core hydrophobic amino acids was assayed by alanine substitution. None of the peptides with a core hydrophobic amino acid substitution were able to compete with wtPALB2 binding. A cancer-associated mutation of the conserved Pro615 to serine resulted in a 5-fold reduction in binding affinity, suggesting an important role of the conserved proline in the FxLP motif for the interaction or conformation of the MRG-binding peptide. Substitution of Asp616 with histidine reduced the affinity by a factor of 2. Although Asp616 does not form any contacts with MRG, it forms a polar interaction with Lys623 (distance 2.8 Å) and can help to position αH2 in the orientation optimal for MRG binding. Surprisingly, substitution of Pro621 in αH2 by threonine increased the binding affinity. Substitution of this proline in the middle of an α-helical turn may increase flexibility and result in a tighter binding of αH2 to the hydrophobic pocket of MRG.

### 3.4. Comparison with Other MRG Complexes

**Figure 6:**
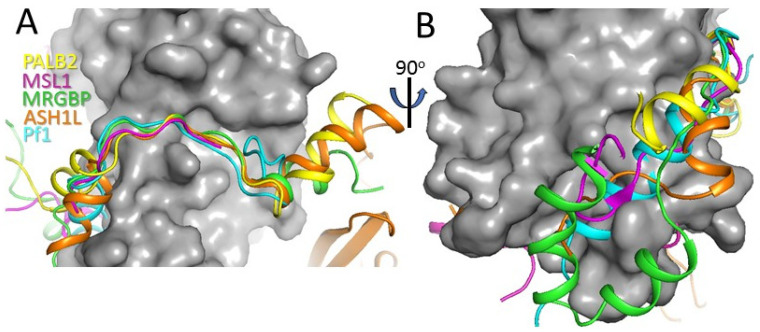
**Comparison of PALB2 peptide conformation with other MRG-binding peptides.** Two orthogonal views of MRG (shown in grey surface representation) complexes with PALB2 (yellow), MSL1 (magenta), MRGBP (green), ASH1L (orange) and Pf1 (cyan) shown in cartoon representations with the middle part of the peptides shown in panel (**A**) and the N-terminal parts in panel (**B**). All structures were superimposed by MRG domain.

MRG domains interact with diverse proteins containing the FxLP motif, including MRGBP [38], Pf1 [37,40], ASH1L [41] and MSL1 [45]. Comparison of the MRG/PALB2 complex with known structures of other MRG complexes demonstrates the involvement of a similar extended, mainly hydrophobic surface area of MRG in peptide binding (Figure 6). Many peptides interact with two extended surface areas of MRG adjacent to the central FxLP-binding cavity (Figure 6A). At the N-terminal end, PALB2 interacts with MRG through αH1 similarly to the α-helices in Pf1 and ASH1L. The same hydrophobic pocket is bound by extended peptides in complexes with MSL1 and MRGBP. In the latter case, additional hydrophobic interactions are formed by an α-helical domain of MRGBP. At the C-terminal end, PALB2 forms αH2, which interacts with MRG in a way that is similar to ASH1L and MRGBP. Correspondingly, PALB2 interacts with the MRG domain with a similar affinity to those reported for MRGBP and Pf1.

## 4. Discussion

Our results show that PALB2 interacts with the MRG15 MRG domain with similar affinity and through the same extended binding interface as other transcription factors. Two α-helices on each side of the FxLP motif bind to the extended surface areas of the MRG domain at the opposite sides of the core FxLP binding cavity, in contrast to a previously predicted model where PALB2 was proposed to interact only through αH2 [38]. Despite extended hydrophobic interactions, single amino acid substitutions of any hydrophobic amino acid in the FxLP(x)_2–3_Ф motif disrupts the interaction. This suggests a multistep interaction mechanism where initial recognition occurs through the FxLP(x)_2_Ф motif, followed by the binding of two α-helices on each side of this central motif. PALB2 competes with other MRG binders for binding to specific MRG domain-containing transcription regulator complexes. Alternatively, it can participate in binding to complexes with multiple MRG domains [38]. It has been reported that a second conserved motif in PALB2, C-terminal to the FxLP motif, also contributes to MRG recognition [18]. This region, termed MBD-II, includes amino acids 724–737, which are more than 100 amino acids away from the FxLP motif (termed MBD-I). We did not test the interaction of the peptide containing this region with MRG15. However, previous studies demonstrated that MBD-II deletion does not prevent interaction in a pull-down assay [18]. It is unlikely that this motif directly participates or significantly contributes to the interaction with the MRG domain, because most of the MRG protein binding interface is already occupied by PALB2^597−630^, which binds the MRG domain with a nanomolar affinity. On the other hand, considering the highly diverse structural flanking regions of FxLP motif of different interactions of other proteins, we cannot rule out an additional extended binding interface of MRG interacting with MBD-II. An additional MRG motif comprised of Ile245 and Leu246 was suggested to serve as a secondary binding site for MBD-II, since the interaction is abrogated by a I245E/L246E double mutant [18]. However, these amino acids form a hydrophobic core of the MRG domain, and a double substitution by negatively charged glutamic acids should destabilize the entire MRG domain structure. Therefore, defects in chromatin localization of PALB2 with deleted MBD-II [18,46] are likely due to its involvement in a non-MRG-related interaction yet to be identified.

The interaction of MRG15 with methylated histones is relatively weak, suggesting that PALB2 localization at actively transcribed genes is mediated through multiple interactions. Indeed, PALB2 has a conserved chromatin interacting motif (ChAM) that is located 250 amino acids upstream of the FQLP motif [47] and interacts with an acidic patch formed by histones H2A-H2B [46]. Both ChAM and MBD contribute to PALB2 chromatin localization. Genome-wide localization analysis suggests a model by which PALB2 and BRCA2 proteins localize to chromatin regions with actively transcribed genes marked by H3K36me3 through the MRG15 interaction [18], protecting the genome from DNA damage due to transcription/replication collision. Upon extensive DNA damage, the PALB2-BRCA2 system is relocated to initiate HR repair through other chromosome regions. In addition, PALB2 indirectly recognizes ubiquitinylated histone H2A through a physical interaction with RNF168 [48,49,50]. Finally, PALB2 directly interacts with DNA [51,52], and mutation of the primary DNA-binding motif strongly attenuates RAD51 foci formation and HR upon DNA damage [53]. Therefore, MRG binding contributes to the multivalent mechanism of PALB2 recruitment to chromatin mediated by the N-terminal DBD, the C-terminal RNF168 binding WD40 domain and the ChAM motif in dividing cells during both normal and stress conditions. This elaborate network of interactions is coupled with a main mechanism of PALB2 recruitment to DNA breaks through the interaction with BRCA1 and reflects a unique function of PALB2 in initiating DNA repair at different chromatin regions under a variety of stress conditions as well as during normal replication. The results of several cell-based experiments and genetic data revealed the importance of each PALB2 chromatin recruitment pathway. However, detailed knowledge of the relationship of these chromatin recruitment mechanisms of PALB2 with each other and with specific cellular events remains absent. 

## 5. Conclusions

The new structural and biochemical data presented in this work describe the mechanism of PALB2 interaction with the MRG domain and suggest that PALB2 competes with transcription MRG-binding factors for the same extended binding interface and interacts with similar affinity. We propose that the MRG-binding motif definition should be extended to include additional conserved hydrophobic interaction as FxLP(x)_2–3_Φ. Results revealed the role of key amino acids in the interaction, a minor effect of cancer-related mutations and suggest that a second conserved sequence motif around amino acids 724–737, previously suggested to interact with MRG, is unlikely to interact with MRG. Our results will help to further delineate a complex mechanism of PALB2 function in chromatin maintenance, to better predict pathogenesis of known and newly identified mutations and to advance personalized treatment of cancer in patients with different genetic backgrounds.

## Figures and Tables

**Table 1:** Data collection and refinement statistics.

Wavelength (Å)	1.54
Resolution range (Å)	30–2.7
Space group	P 21 2 21
Unit cell dimensions a, b, c (Å) a, b, g (Å)	54.08; 60.16; 131.85 90.00; 90.00; 90.00
Total reflections	410,291
Unique reflections	12,298 (603) *
Multiplicity	4.7 (4.6)
Completeness (%)	98.7 (99.5)
Mean I/sigma (I)	10.2 (1.7)
Wilson B-factor	28.32
R-merge	0.077 (0.65)
R-pim	0.065
CC1/2	0.995 (0.675)
Refinement resolution range (Å)	29.7–2.7
Reflections used in refinement	12,252 (1180)
Reflections used for R-free	1228 (131)
R-work	0.226 (0.289)
R-free	0.297 (0.378)
Number of non-hydrogen atoms	3153
RMS (bonds)	0.009
RMS (angles)	1.269
Ramachandran favored (%)	96.8
Ramachandran allowed (%)	3.2
Ramachandran outliers (%)	0.0
Clashscore	8.4
Average B-factor	33.0

* Numbers in parentheses show corresponding values for high resolution shells. 2.70–2.75 Å was used for data scaling and 2.70–2.80 Å was used for the refinement statistics.

## Data Availability

Coordinates of the refined MRG-PALB2 structure and experimental structure factors are deposited to PDB with PDB ID 7S4A.
